# New Materials Based on Polyvinylpyrrolidone-Containing Copolymers with Ferromagnetic Fillers

**DOI:** 10.3390/ma15155183

**Published:** 2022-07-26

**Authors:** Oleksandr Grytsenko, Ludmila Dulebova, Emil Spišák, Bohdan Berezhnyy

**Affiliations:** 1 Department of Chemical Technology of Plastics Processing, Lviv Polytechnic National University, 12, St. Bandera Str., 79013 Lviv, Ukraine; oleksandr.m.grytsenko@lpnu.ua (O.G.); bohdan-volodymyr.berezhnyi.mnkhtm.2021@lpnu.ua (B.B.); 2 Department of Technologies, Materials and Computer Aided Production, Technical University of Kosice, 74 Mäsiarska, 04001 Kosice, Slovakia; emil.spisak@tuke.sk

**Keywords:** hydrogels, composite hydrogels, ferromagnetic fillers, magnetic hydrogels, copolymers, polyvinylpyrrolidone, 2-hydroxyethylmethacrylate

## Abstract

The article investigates the peculiarities of the effect of ferromagnetic fillers (FMFs) of various natures (Ni, Co, Fe, FeCo, SmCo_5_) on the formation of the structure and properties of 2-hydroxyethylmethacrylate (HEMA) with polyvinylpyrrolidone (PVP) copolymers. The composites were characterized using FTIR-spectroscopy, SEM, DMTA, magnetometry of vibrating samples, specific electrical resistivity studies, and mechanical and thermophysical studies. The formation of a grafted spatially crosslinked copolymer (pHEMA-gr-PVP) was confirmed and it was established that the FMF introduction of only 10 wt.% into the copolymer formulation increased the degree of crosslinking of the polymer network by three times. The surface hardness of composites increased by 20–25%. However, the water content decreased by 16–18% and lay within 42–43 wt.%, which is a relatively high number. The heat resistance of dry composites was characterized by Vicat softening temperature, which was 39–42 °C higher compared to the unfilled material. It was established that the obtained composites were characterized by a coercive force of 200 kA × m^−1^ and induction of a magnetic field at the poles of 4–5 mT and 10–15 mT, respectively. The introduction of FMF particles into pHEMA-gr-PVP copolymers, which, in the dry state, are dielectrics, provides them with electrical conductivity, which was evaluated by the specific volume resistance. Depending on the FMF nature and content, as well as their orientation in the magnetic field, the resistance of filled materials could be regulated within 10^2^–10^6^ Ohm·m. Therefore, the modification of HEMA with PVP copolymers by ferromagnetic fillers of various natures provides the possibility of obtaining materials with unique predicted properties and expands the fields of their use, for instance as magnetic sorbents for various applications, as well as the possibilities associated with their being electrically conductive materials that can respond by changing of electrical conductivity, depending on various factors.

## 1. Introduction

The processes of modification of polymeric materials with metal fillers of different natures are the subject of numerous studies, the result of which is the creation of new materials [1] with unique properties for different fields of science [2] and practice [3]. Nowadays, metal-filled hydrophilic polymers and hydrogels based on them are a new advanced class of polymer composite materials [4,5]. Polymer hydrogels are characterized by a unique porous structure [6] with the presence of hydrophilic functional groups, which provides swelling of the polymer matrix in water and other polar solvents, as well as high permeability to dissolved low molecular weight substances [7]. Composite metal-filled polymer hydrogels consist of at least two components, a hydrogel matrix and a fine metal filler. Basically, the hydrogel matrix provides strength, resilience, sorption capacity and processability of the composite obtaining process [8], while the introduced filler particles provide many functional capabilities, such as redox [9], catalytic [10], magnetic [11], optical [12], electrically conductive [13], bactericidal and antifungal activities and properties [14] among others. The combination of the properties of the hydrogel matrix and metal filler of different natures creates unique synergistic effects that are absent in the individual components [15]. In many cases, it is necessary to ensure a change in the behavior of the hydrogel depending on the action of external factors, such as temperature, pressure, pH of the medium, etc. One of the remotely controlled methods of the hydrogel state is to integrate the sensitivity to magnetic fields [16].

Hydrogels that are magnetic and those that are sensitive to the magnetic field are usually obtained by introducing into ferromagnetic fillers (FMFs), such as Fe [17], Ni [18], Co [19], Fe_2_O_3_ [20], Fe_3_O_4_, into the hydrophilic polymer matrix [21]. Magnetic properties, in combination with biocompatibility and sorption capacity, provide unique functional capabilities to composite hydrogels, which are used in a wide range of practical applications in the biomedical engineering field [22], including tissue engineering [23], drug delivery and release [24], cancer therapy [25], biosensors [26], etc. FMF-filled hydrogels, due to their unique properties and ability to respond to external stimuli of various natures, are ideal materials for the manufacture of detectors and sensors [16]. On the basis of magnetic hydrogels, polymer-immobilized catalysts are obtained, which possess high selectivity and stability of action, and are able to accelerate the reactions of decomposition, hydrogenation, oxidation, isomerization, etc. [27,28]. Magnetic hydrogel adsorbents are mainly used for heavy metal removal, separation, destruction and adsorption of oil, dyes, toxic organic compounds, several biomolecules and drugs [20]. FMF-filled hydrogels are used to clean the inner surface of oil and gas pipelines, as well as to apply anti-corrosion solutions to the inner surface of pipelines before their operation [29]. The properties of magnetic hydrogels (e.g., magnetic response) rely on several factors, including the type of hydrogel and FMF used, the hydrogel and FMF concentration, and the size and distribution of the FMF within the hydrogels [22].

Methods of obtaining hydrogel magnetic composites are constantly being technologically improved and refined. For example, a promising method is polymerization with simultaneous precipitation of metal, which consists in combining the processes of reduction of metal ions and the synthesis of the polymer matrix [30]. At the same time, one of the main factors influencing the choice of the field of use of FMF-filled hydrogels is the control over the size and shape of the filler particles [31]. Therefore, hydrogels filled with nanoscale ferromagnets, often referred to as nano heterogeneous composite ferrogels or nanocomposite ferrogels, are of particular interest at present [32]. However, methods for producing hydrogels filled with ferromagnet nanoparticles are usually technologically complex, especially on an industrial scale. For example, the filling, during which FMF particles are formed in the polymer matrix by one of the chemical methods, is at least a two-step process [10] and is recommended to be performed when the hydrogel is in the form of a thin film or balls with a diameter of nano- and micro-sizes. During the obtainment of composite hydrogels by the method of polymerization with simultaneous precipitation of the metal, by-products of the reduction reaction are isolated, which affects the increase of the macro-porosity of the polymer matrix [18]. This, in turn, leads to a significant reduction in the strength and resilient characteristics of the composite. At the same time, methods that include the stage of chemical reduction of metal ions (both in the polymer network and during polymerization with simultaneous reduction) require the stage of purification (washing) of hydrogels from by-products of the reduction reaction. A significant disadvantage is the use of toxic reducing agents, which often limits the use of metal-filled hydrogels, especially in the medical field.

For some applications, such as the obtainment of magnetic sorbents or oil and gas pipe cleaners, filling the composite with a nanoscale filler is not always a basic requirement. A disadvantage of such particles may be that the magnetic force is small due to the small volume, so that viscous forces dominate and magnetic separations can take a long time (tens of minutes). This explains the interest in using larger magnetic particles (typically 0.2–5 μm in diameter) for some applications. Such microparticles demonstrate strong response to an external magnetic field. Also, they possess remanent magnetization and coercivity [29]. Therefore, in some cases, it is necessary to use more technologically economical and simpler methods. Currently, there is insufficient information in scientific sources concerning the filling of polymer hydrogels with micro-sized powders of metals. Due to the spatially crosslinked structure of hydrogels, the introduction of prepared FMF particles into their composition is possible by a limited number of methods, namely, during the formation of the hydrogel matrix by polymerization or by precipitation from solution [33,34]. Filling during polymerization is the simplest method from a technological point of view. During the obtainment of hydrogel composite prepared FMF particles are used, which are dispersed in a monomer (or reaction composition) followed by polymerization [33,34]. The technological design of the method does not require expensive equipment. However, the filling during polymerization is accompanied by sedimentation of the filler particles. Sedimentation causes inhomogeneity of the composite in volume and, as a consequence, the appearance of anisotropy of its properties. Therefore, in each case, depending on the nature of the reaction composition and FMF, the dispersity of FMF, it is necessary to make technological decisions to obtain the composite material with the necessary structure and set of properties. The solution to this problem may be the structuring of FMF during the polymerization process of the filled composition in a magnetic field [35]. Also, at the polymerization stage, the filler is introduced at the end of the pot life time of the reaction composition (the time when the composition remains fluid) [36]. During polymerization, high-reactive compositions having a polymerization process of short duration are used for filling; for example, polymer-monomer compositions based on polyvinylpyrrolidone (PVP) with 2-hydroxyethylmethacrylate (HEMA) in the presence of metal ions of variable oxidation state [37].

Unique technological and operational characteristics of copolymers HEMA with PVP (pHEMA-gr-PVP) are provided by PVP [38]. An important characteristic of PVP is its ability to form complexes with low molecular weight substances [39], including monomers [40]. The role of PVP complexes with vinyl monomers is particularly noteworthy [41]. Specifically, this ability has a significant impact on the kinetics of polymerization of HEMA in the presence of PVP (through the formation stage of a complex with charge transfer) and the formation of the polymer matrix structure [42]. At the same time, the use of PVP opens up additional opportunities for modification of various substances [43,44] and improvement of technologies for obtaining new functional materials with a set of unique properties [45,46].

The work [42] presents the study results of the structure and properties of pHEMA-gr-PVP copolymers and their hydrogels obtained by bulk polymerization in the presence of ferrous sulfate (II). The course of grafting polymerization of HEMA on PVP with the formation of a crosslinked copolymer was confirmed by chemical analysis, IR spectroscopy, TGA and DTA. It was established that the directed change of the original composition formulation is an effective way to regulate the structure (copolymer formulation and network density), and, accordingly, the physico-mechanical, transport-diffusion and thermophysical properties of copolymers. As the PVP content in the original composition increases, its grafting efficiency and the crosslinking density of the polymer network decrease. In addition, hardness, heat resistance and sorption capacity of the copolymers in the dry state increase, as well as ion permeability and elasticity in the swollen state, but the tensile strength of the swollen hydrogels decreases. Copolymerization of HEMA with PVP under the action of FeSO_4_ occurs at high rate in air at room temperature without additional vacuuming, which greatly simplifies the technology of obtaining copolymers and hydrogels based on them, including those filled with FMF particles.

At the same time, the prospects of practical uses of pHEMA-gr-PVP copolymers filled with FMF (FMF/pHEMA-gr-PVP) provide such characteristics of the polymer matrix as large porous structure, swelling ability, sorption capacity of low molecular weight compounds, resistance to aggressive mediums, mechanical strength and elasticity [38,47].

Certainly, the modification of pHEMA-gr-PVP copolymers with ferromagnetic fillers will be accompanied by synergistic effects, the consequence of which is the acquisition of unique properties by composite materials. It is possible to predict that the presence of a metal surface at the synthesis stage of the copolymers will affect the course of the polymerization process, the formation of the polymer matrix structure and, hence, the properties of the composite. Taking into account the processability of HEMA/PVP compositions, their high reactivity in the presence of FeSO_4_, the unique properties of pHEMA-gr-PVP copolymers, as well as the lack of works related to the study of composites based on FMF/pHEMA-gr-PVP copolymers, research in this direction is relevant. Knowledge of the peculiarities of the effect of FMF on the structure and properties of pHEMA-gr-PVP copolymers will open up prospects of creating new composite materials based on them and expand the fields of their use.

The aim of the work was to investigate the features of the influence of fine powders of ferromagnetic fillers with different natures on the formation of the structure and on the properties of composite pHEMA-gr-PVP copolymers.

## 2. Materials and Methods

### 2.1. Materials

The following substances were used: 2-hydroxyethylmethacrylate (Sigma Chemical Co., Saint Louis, MO, USA), which was purified and distilled in a vacuum (residual pressure = 130 N/m^2^, TB = 351 K); polyvinylpyrrolidone (AppliChem GmbH, Darmstadt, Germany) of high purity with MM 12,000, which was dried at 338 K in a vacuum for 2–3 h before use; iron (II) sulfate of p.a. grades. For the preparation of the reaction composition, considering its processability, HEMA in the amount of 70–80 mass parts and PVP in the amount of 20–30 mass parts were used. The lower limit of the PVP content was driven by the fact that its lower content significantly increases the hardening time of compositions, especially metal-filled compositions, which were hardened without additional polymerization initiators. The upper limit was due to technological complications. A higher content of PVP increases the duration of its dissolution in HEMA, and increases the viscosity of the composition, which becomes difficult to dose. Fine powders of metals (Ni, Co, Fe) and their alloys (FeCo, SmCo_5_) with a particle size of 10–50 μm were used as FMF for research.

### 2.2. Synthesis Technique of pHEMA-gr-PVP Copolymers and FMF-Filled Composites Based on Them

Copolymerization of HEMA with PVP was carried out in bulk with the presence of iron sulfate (II) at room temperature [42]. The calculated amount of PVP and FeSO_4_ were weighed according to the given formulation. HEMA was dosed by the volumetric method. Into 1/3 part of the HEMA amount, required for polymerization, a certain amount of FeSO_4_ (0.05 wt.%) was dissolved. The required amount of PVP was dissolved in the rest of the HEMA content. Stirring was performed at room temperature until there was complete dissolution of FeSO_4_ and PVP in HEMA. Solutions of FeSO_4_ and PVP in HEMA were mixed to obtain a homogeneous mixture, without insoluble agglomerates and mechanical inclusions, which was processed by the casting method into a polymerization mold. The obtained products were rinsed in distilled water until there was complete removal of unreacted HEMA and PVP [42].

Polymerization of HEMA with PVP compositions filled with FMF powders was performed with the presence of iron (II) sulfate and without FeSO_4_. By mixing the required amounts of HEMA with PVP for polymerization, a polymer-monomer composition (PMC) was obtained, to which the required amount of filler powder was added. If necessary, FeSO_4_ was previously dissolved in HEMA. The obtained PMC with the filler was stirred occasionally during the time τ < τ_p.l._ (τ_p.l._—the pot life time of the composition—the time during which the reaction composition retains fluidity), after which it was dosed into a polymerization mold. The polymerization was carried out at room temperature for 0.3–1 h (depending on the composition formulation, nature and amount of FMF).

### 2.3. Measurements and Characterization

#### 2.3.1. Investigation of Polymerization Kinetics of HEMA/PVP Compositions

The kinetics of polymerization on the initial stages (polymer yield up to 10 wt.%) was investigated by the dilatometric method by changing the volume of the reaction mixture. A 5 cm^3^ glass dilatometer with uniform stirring was used for the experiment. The change in volume of the reaction mixture during polymerization was registered optically with a cathetometer [48]. The polymerization reaction rate (*V*) was calculated with monomer conversion of 5–10% from the following dependence:

(1) $$V=\frac{C_{M}\cdot A}{\tau },$$

where: *C_M_*—Monomer concentration, mol/L; *A*—Monomer conversion; *τ*—Polymerization time, s.

#### 2.3.2. Standard Methods of Instrumental Research

Attenuated total reflectance Fourier transform infrared (ATR FTIR) spectra were obtained using a PARAGON 1000 FTIR spectrometer equipped with a single-horizontal Golden Gate ATR cell (Perkin-Elmer, Waltham, MA, USA). Samples were used in the form of pellets. The spectra were recorded after 16 scans, at a resolution of 4 cm^−1^, within the range of 500 to 4000 cm^−1^. The morphology of samples in the dry state was studied by scanning electron microscopy (SEM). Research was done using a scanning electron microscope-microanalyzer PEMMA-102-02 (JSC “SELMI”, Sumy, Ukraine). The range of accelerating voltage change was 0.2–40 keV, the range of magnification change was 10–300,000, and resolution was no more 5.0 nm.

#### 2.3.3. PVP Grafting

The method of determining PVP was based on the formation of its colored complex with iodine. To determine the PVP that entered into the grafting reaction with the monomer, the amount of unbounded PVP in the copolymer was found by studying its water extracts according to the procedure described in [49].

#### 2.3.4. The Molecular Weight between Crosslinks in Polymer Network

The molecular weight between crosslinks in the polymer network (*M_C_*, kg/mol) was determined using the Flory-Rehner method [50].

#### 2.3.5. Physico-Mechanical Characteristics of FMF/pHEMA-gr-PVP Copolymers

Dynamic mechanical characteristics, in terms of the tangent of an angle of mechanical losses *tgδ* and the storage modulus *G*′, were studied by the method of dynamic mechanical thermal analysis (DMTA) with the help of a rheometer, Rheometric ARES (Advanced Rheometric Expansion System) (Rheometric Scientific, Inc., Piscataway, NJ, USA), using resilient oscillating deformation with a frequency of 1 Hz in the temperature range 25–200 °C and a heating rate of 3 °C/min. The surface hardness of dry samples (with diameters Ø 12 ± 0.1 mm and height h = 5 ± 0.01 mm) was found according to the conic yield point in a Höppler consistometer (VEB Pruefgeraetewerk, Medingen, Dresden, Germany) at 293 °K by pressing a steel cone with a vertex angle of 58°08′ into a polymeric specimen under a load of 5.0 kg for 60 s [18]. Boundary water adsorption (*W*, wt.%) and hydrogel swelling factor (k) were determined according to the procedures described in [37]. Deformation and elastic characteristics of samples in swollen state, such as hardness number (*H*, MPa), elasticity index (*E*, %) and plasticity index (*P*, %), were determined according to the procedures described in [37] and ASTM D2240-15 “Standard Test Method for Rubber Property—Durometer Hardness”.

#### 2.3.6. Thermophysical Properties

Determination of heat resistance of copolymers was carried out according to the method ISO 306:2013 “Plastics—Thermoplastic materials—Determination of Vicat softening temperature”, which consisted of determining the temperature at which a standard indenter, with a flat bottom surface (Ø 1.128 ± 0.008 mm), was pressed under the action of the load to a depth of 1 mm in the test sample, which was heated at a constant speed. Studies were performed by using a Höppler consistometer [42].

Thermomechanical studies were performed according to ISO 11359-1:1999 “Plastics. Thermomechanical analysis (TMA). Part 1. General principles”, the method of which consists in determination of the polymer sample deformation under the action of a permanent load, which is registered as a function of temperature in the form of a thermomechanical curve (TMC). The TMC was obtained on a Höppler consistometer by recording the deformation of the sample (4 mm thick tablets) due to the action on the rod (area 23.7 mm^2^) of a load of 5.0 kg. The initial temperature of the study was 293 K. Subsequent deformation measurements were carried out after increasing the temperature every 3 °C. The heating rate was set to ≈1 °C/min. The deformation of the samples (*ε*) was determined by the formula:

(2) $$\varepsilon =\frac{\Delta l}{h}\cdot 100\%=\frac{l-l_{0}}{h}\cdot 100\%,,$$

where: *l*_0_—Load-free indicator readings (before lowering the rod), mm; *l*—Indicator readings after 10 s of holding under load, mm; *h*—Height of a tablet, mm.

#### 2.3.7. Conductivity

The conductivity characteristics of FMF/pHEMA-gr-PVP composites were estimated by the specific volume resistivity (*ρ_v_*, Ω···m) according to ASTM D4496-21 “Standard Test Method for D-C Resistance or Conductance of Moderately Conductive Materials”. Samples of cylindrical shape with a diameter of (12 ± 0.1) mm and a height of (5 ± 0.1) mm were used for *ρ_v_* study. Specific volume resistivity was calculated as [18]:

(3) $$\rho_{v}=\frac{R_{v}\cdot S}{h},$$

where: *R_v_*—Volume electrical resistance, Ω; *S*—Square of the sample, m^2^; *h*—Sample thickness, m.

Samples of composites obtained by polymerization filling in a magnetic field were investigated in the form of a cube with an edge size of 5 mm. The resistance of such samples was measured in two directions, parallel and perpendicular to the magnetic field lines (FMF particle chain construction).

#### 2.3.8. Magnetic Properties

Magnetic characteristics (specific saturation magnetization, coercivity) of FMF/pHEMA-gr-PVP composites were determined using a magnetometer MPMS-XL5 (Quantum Design, San Diego, CA, USA) with a vibrational sample. For analysis, samples of a cylindrical shape with a diameter of 8 mm and height of 3 mm were used, and a standard carbonyl iron was used [18].

## 3. Results and Discussion

### 3.1. Features of FMF Effect on the Formation of the Structure of HEMA-gr-PVP Copolymers

**Figure 1:**
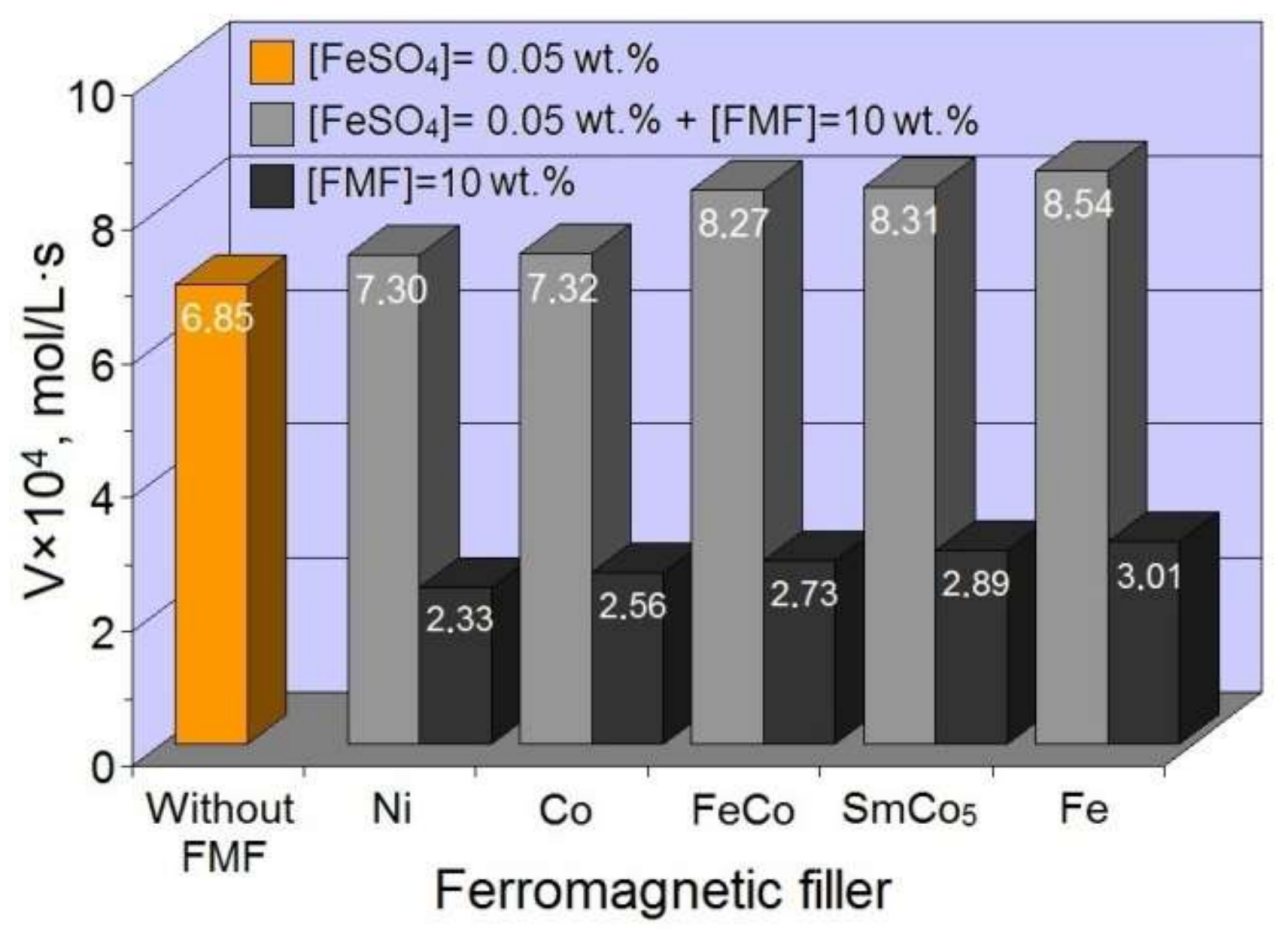
Effect of FMF nature on the initial polymerization rate of HEMA/PVP compositions (HEMA:PVP = 80:20 mass parts).

During the filling of HEMA/PVP compositions with FMF powders at the polymerization stage, it was proved that the copolymerization of HEMA with PVP occurred at a higher rate than the polymerization only under the influence of FeSO_4_ (Figure 1).

At the same time, it was found that although the polymerization of compositions containing FMF occurred at a lower rate, it occurred without additional catalysts, which indicated active participation of the filler surface in initiating of the polymerization (Figure 1) [36]. Therefore, the synthesis of FMF/pHEMA-gr-PVP copolymers could be carried out in two ways, by polymerization with simultaneous filling and by polymerization filling. Polymerization with simultaneous filling consists in mechanical mixing of the original composition with the filler and its subsequent hardening under the action of initiators or catalysts (in this case, in the presence of FeSO_4_). Polymerization filling differs, because the synthesis of the polymer matrix occurs without using an additional initiator or catalyst. The method consists in polymerization in the presence of metal particles, which, on the one hand, play the role of a filler, and on the other, act as the catalyst of the polymer formation reaction. Polymerization filling is currently a relatively new direction in the synthesis of metal-filled hydrophilic polymers and hydrogel materials based on them [51].

**Figure 2:**
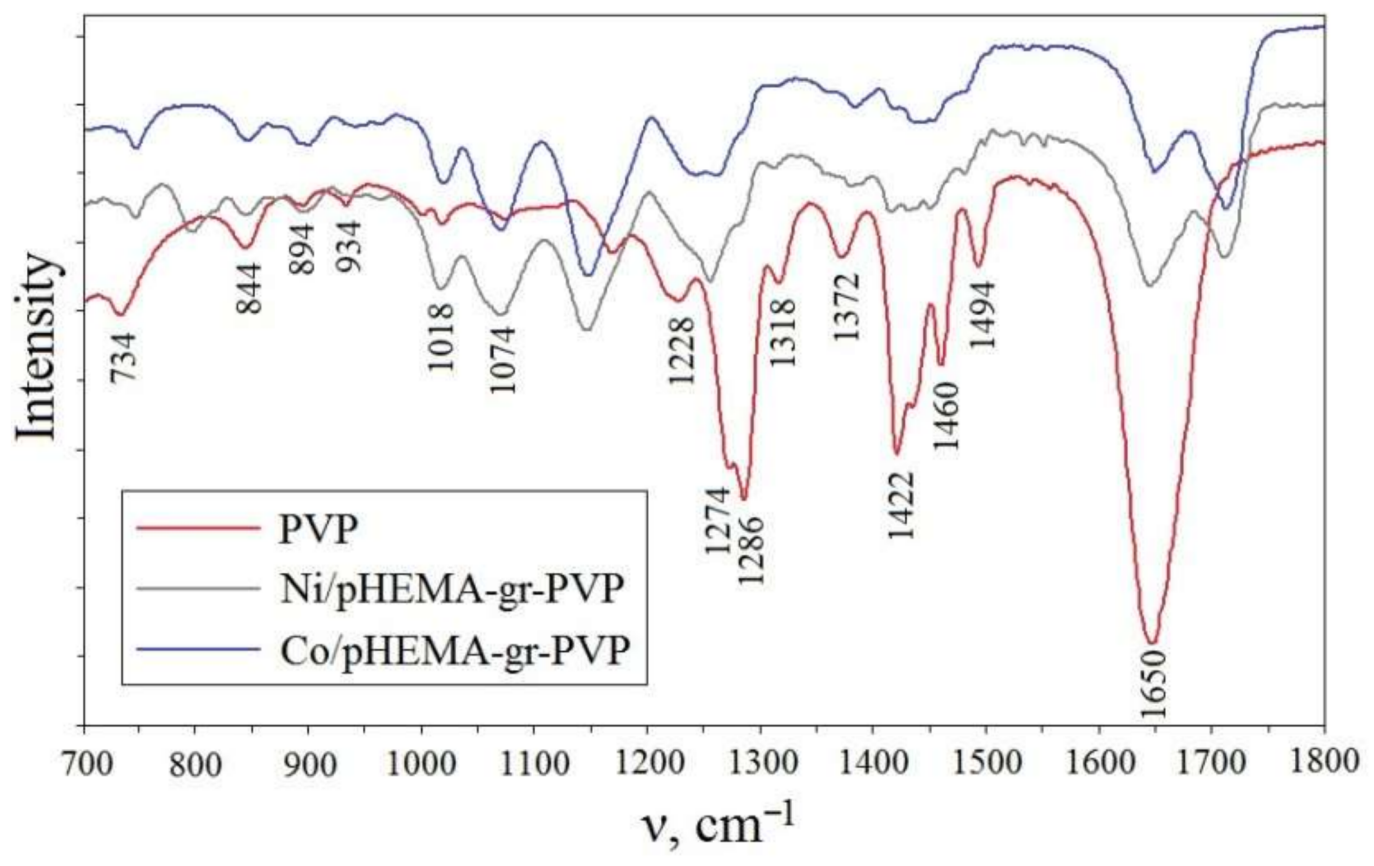
ATR FTIR spectra of PVP and FMF/pHEMA-gr-PVP composites (HEMA:PVP = 80:20 mass parts, FMF content—[FMF] = 10 wt.%).

During the polymerization filling, the introduction of the filler into the polymer was carried out directly at the stage of its synthesis and the metal surface present in the zone of the polymerization reaction affected the whole process of polymer formation. Naturally, the introduction of filler in pHEMA-gr-PVP copolymers at the stage of synthesis affected the formation of their structure, and hence their properties. In order to define the influence of the surface of the metal filler on the formation of the structure of the polymer matrix, the copolymerization of HEMA with PVP was carried out without an additional initiator FeSO_4_. IR spectra of PVP, HEMA and copolymers of PVP with HEMA obtained in the presence of FMF are presented (using the example of Ni and Co) in Figure 2.

**Scheme 1:**
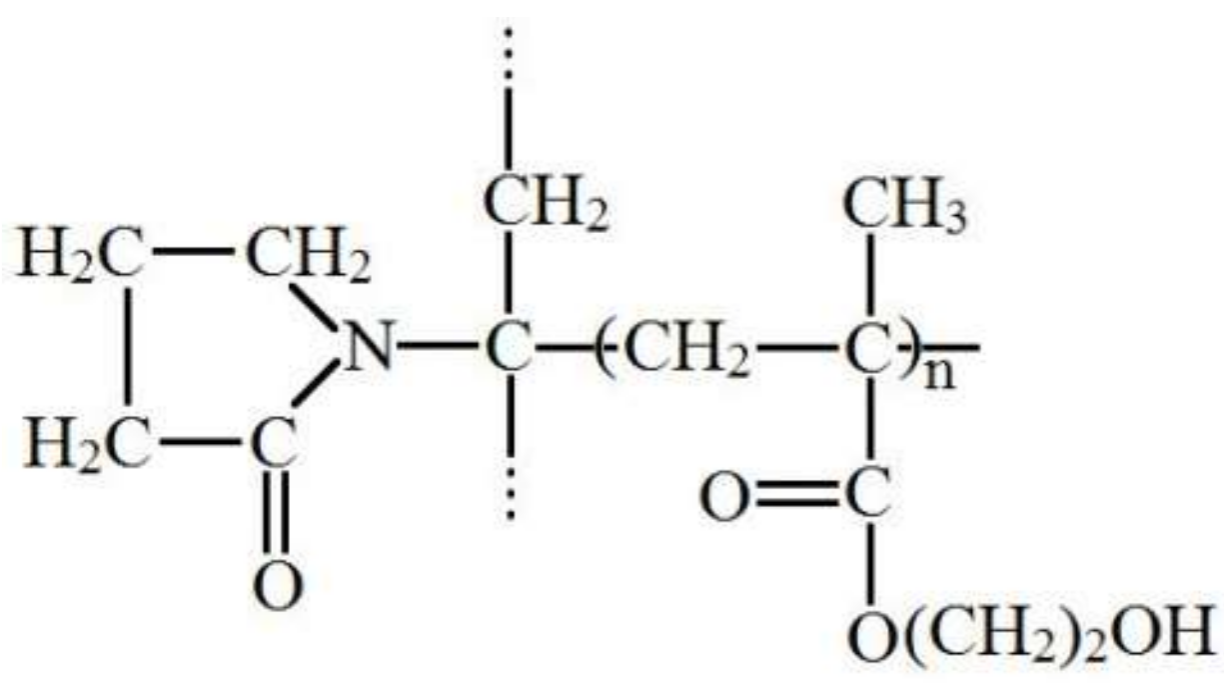
The structure of pHEMA-gr-PVP copolymer [42].

Analysis of the obtained spectra of copolymers extracted with water until complete removal of unreacted components showed that, in the spectra of filled copolymers, the characteristic bands of PVP were presented; in particular, in the regions of 844 cm^−1^, 1170 cm^−1^; 1320 cm^−1^; 1460 cm^−1^, 1650 cm^−1^ [52]. This fact indicated the presence of PVP units in the copolymer. The hydrogen abstraction from tertiary carbon during the graft polymerization was indicated by a change in the intensity of adsorption band in the range 1318–1320 cm^−1^ (Figure 2), which decreased significantly in the case of transition from PVP to extracted filled copolymer and was characteristic for deformation vibrations of C-H group of the carbon chain [42,53]. Moreover, the IR spectrum of the metal-filled copolymer showed a significant decrease in the intensity of the characteristic PVP band in the range of 1372 cm^−1^, which indicated wagging vibrations of the CH_2_-CH bond of polyvinylpyrrolidone, proving the PVP tertiary carbon participation in the grafting reaction (Scheme 1) [42].

**Scheme 2:**
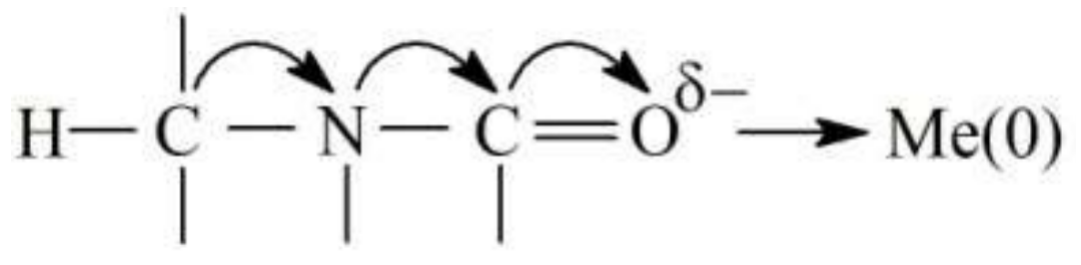
Weakening of the hydrogen bond with the tertiary carbon atom of the PVP main chain.

**Figure 3:**
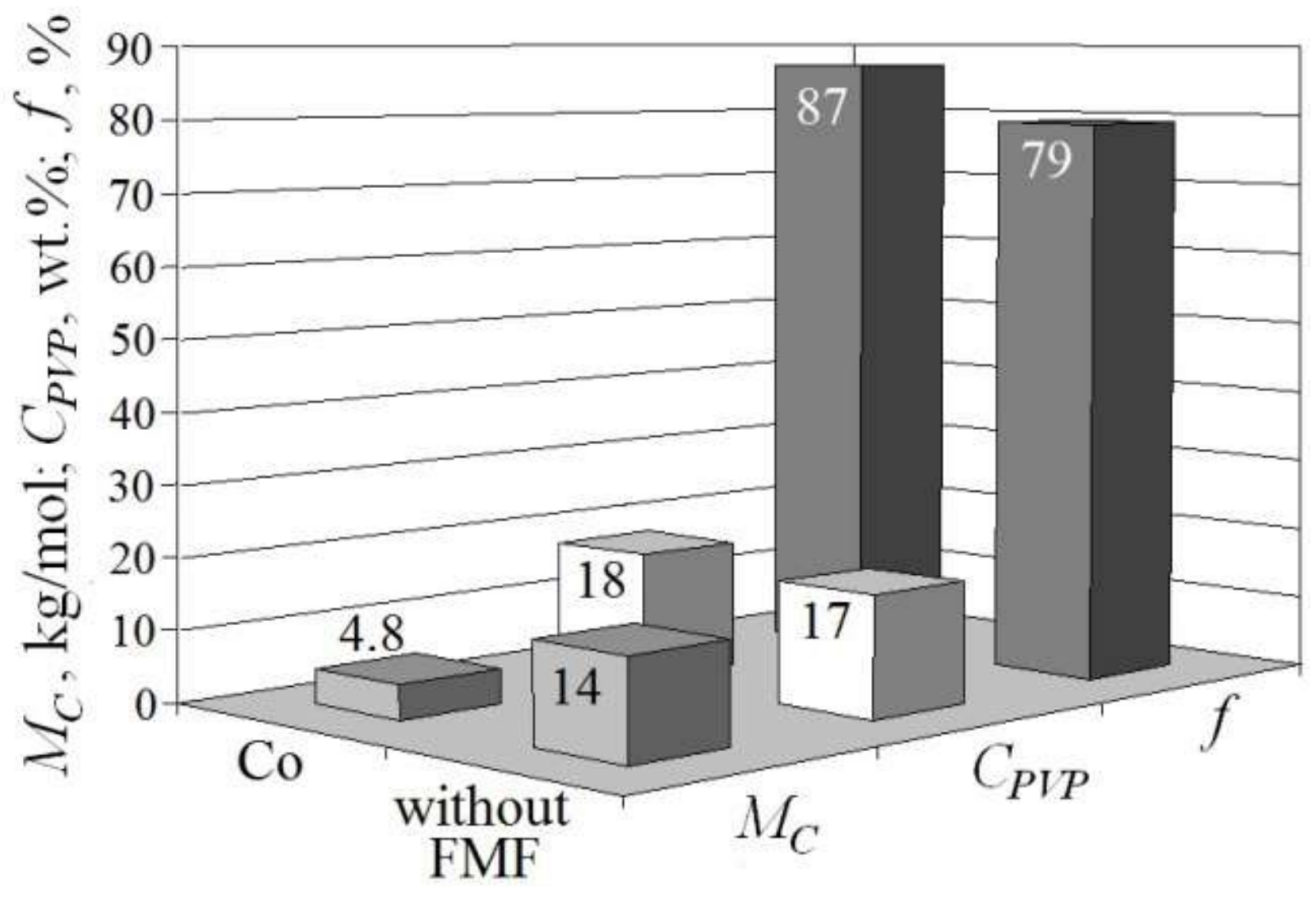
The effect of FMF on structural parameters of pHEMA-gr-PVP copolymers (HEMA:PVP = 80:20 mass parts, [Co] = 10 wt.%): grafting efficiency (*f_PVP_*), PVP content (*C_PVP_*) and molecular weight between crosslinks in polymer network (*M_C_*).

The group -C-N= of pyrrolidone cycle was characterized by a significant deviation in the direction of vibrating frequencies decrease of its characteristic bands (from 1286 cm^−1^ to 1260 cm^−1^), which confirmed its direct participation in physical interaction with FMF particles. This change indicated a weakening of the C-H bond, due to the enhancement of the mesomeric effect by the metal (Scheme 2):

Previous studies [38,42] have shown that during the polymerization of HEMA with PVP, grafted spatially crosslinked copolymers are formed. In our case, polymerization system was hetero-phasic, where the solid phase FMF was characterized by a certain activity that affected the polymerization process (Figure 1) and, accordingly, the formation of the polymer matrix structure. Additional confirmation of the formation of the grafted copolymer were the analysis results of the aqueous extract from obtained composite. Due to the fact that not all PVP underwent the grafting reaction, there was a discrepancy between the PVP content in the original composition and in the copolymer composition [38,42]. In order to determine the effect of FMF on the amount of PVP involved in the grafting reaction, the following parameters were calculated: the efficiency of PVP grafting (*f_PVP_*, %) and its content in the copolymer (*C_PVP_*, wt.%). To characterize the polymer network, the degree of its crosslinking (*ν*, mol/kg) was determined, which was evaluated by the molecular weight between crosslinks in the polymer network (*Mc*, kg/mol) (Figure 3).

**Figure 4:**
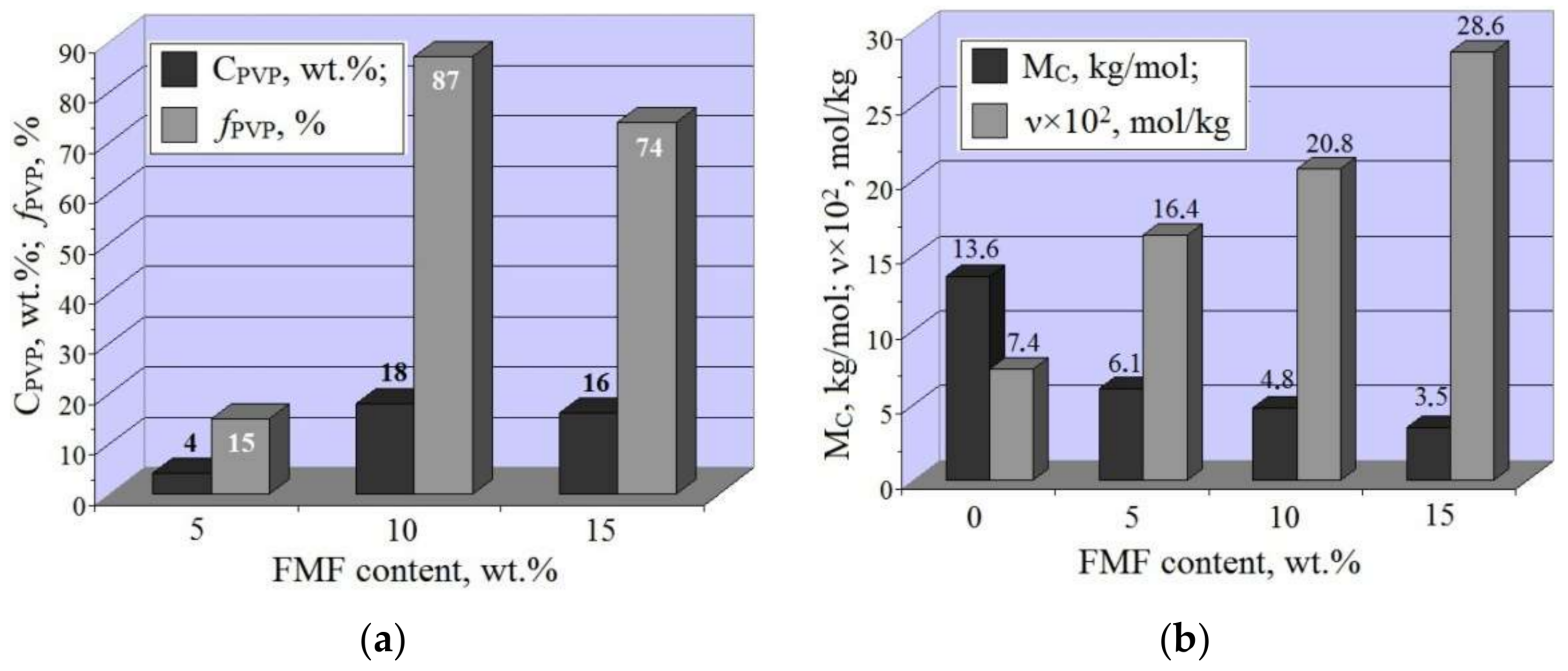
The effect of cobalt powder content on structural parameters of pHEMA-gr-PVP copolymers (HEMA:PVP = 80:20 mass parts, [FMF] = 10 wt.%): (**a**) grafting efficiency (*f_PVP_*) and PVP content (*C_PVP_*), (**b**) molecular weight between crosslinks in polymer network (*M_C_*) and the degree of crosslinking (*ν*).

For comparison, the structural parameters of the polymer network for unfilled copolymer obtained by copolymerization of HEMA with PVP in the presence of Fe^2+^ ions were also presented [42]. The presence of metal particles is the reason for the formation of copolymers with a higher degree of crosslinking, which indicates the formation of additional spatial network, due to the formation of physical nodes, the elements of which can be metal particles. The increase of metal particles content in the original composition led to changes in the efficiency of PVP grafting and its content in the copolymer by extreme dependence (Figure 4a).

**Figure 5:**
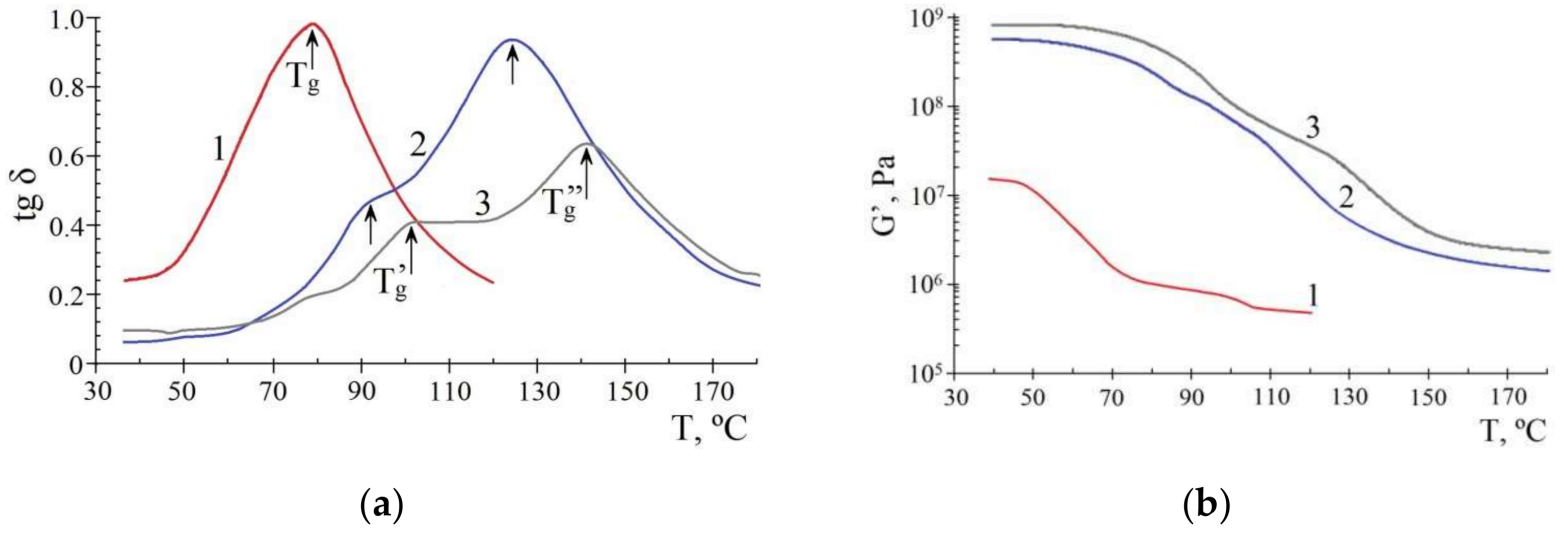
The effect of FMF on the tangent of an angle of mechanical losses *tgδ* (**a**) and the storage modulus *G’* (**b**) of pHEMA-gr-PVP copolymers (HEMA:PVP = 80:20 mass parts; [Me] = 10 wt.%; *T_g_’*—Glass transition temperature in volume, *T_g_”*—Glass transition temperature at the phase boundary): 1—Unfilled copolymer; 2—Co; 3—Ni.

At the same time, the *M_C_* decreased (Figure 4b), i.e., the degree of crosslinking of the polymer network increased, which could be interpreted as the participation of metal particles in the formation of additional physical fluctuation nodes. The presented results confirm the effect of FMF on the course of formation of the polymer matrix structure. However, this effect can occur only on the surface of metal particles, which is a consequence of the formation of a surface-active layer other than the volume of the reaction mass. Obviously, this is the reason for the difference in the rate and nature of the formation of the structure of the copolymer in the surface layer of the metal particle and in the volume of the composition. This phenomenon can be confirmed by the data of a dynamic mechanical thermal analysis (DMTA). DMTA is especially relevant for polymers filled with metal particles, as it characterizes the effect of the filler on the change in the mobility of macromolecules in the boundary surface layers. At the interface between the phases of polymer and metal particle, the nature of the relaxation processes changes, because, due to adsorption phenomena in the boundary layers of filled polymer systems, changes occur in the molecular mobility of system components. The results of the DMTA are presented in the form of curves of temperature dependence of the tangent of an angle of mechanical loss (*tgδ*) and the storage modulus (*G*′, Pa) in Figure 5.

**Figure 6:**
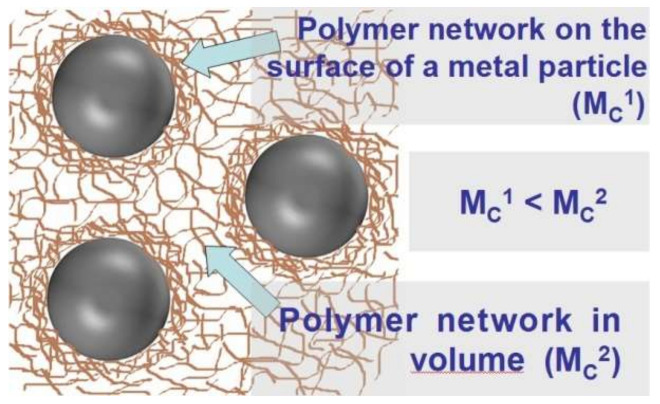
Scheme of polymer matrix structure formation in the presence of a metal surface.

The temperature transitions of filled copolymers (Figure 5, curves 2, 3), compared with unfilled (Figure 5, curve 1), were significantly shifted toward higher temperatures, which was characteristic of both changes in *tgδ* and changes in *G’*. The glass transition temperature (*T_g_*) is the limit of the physical transition of the polymer phase. The value of *T_g_* of the polymer corresponded to the peak on the dependence curves *tgδ* = *f*(*T*) (Figure 5a). A characteristic feature of the studied materials was the presence of two maxima on the temperature dependence of the tangent of an angle of mechanical losses on temperature, which indicated the existence of two areas, where copolymer with different structure was formed, namely in the interfacial layer on the surface of the metal particle and in the volume (Figure 6).

The obtained dependences provided the possibility of comparing the changes in the tangent of an angle of mechanical losses (Figure 5a) and the storage modulus (Figure 5b) of copolymers and to draw a conclusion about the effect of metals on the formation of the polymer matrix. It was found (Figure 1) that the polymerization rate of HEMA/PVP compositions in the presence of Co powder was slightly higher compared to the composition filled with Ni powder. The same regularity was observed for the changes in the intensity of relaxation peaks on the dependences *tgδ = f*(*T*). Therefore, it followed that, with increasing activity of metal in polymerization, molecular mobility and the ability to deform the copolymer increased as well. Meanwhile, a decrease of *G*′ indicated an increase in the resilience of the system.

### 3.2. Properties of FMF/HEMA-gr-PVP Composites

#### 3.2.1. Sorption Capacity of FMF-Filled pHEMA-gr-PVP Copolymers

**Figure 7:**
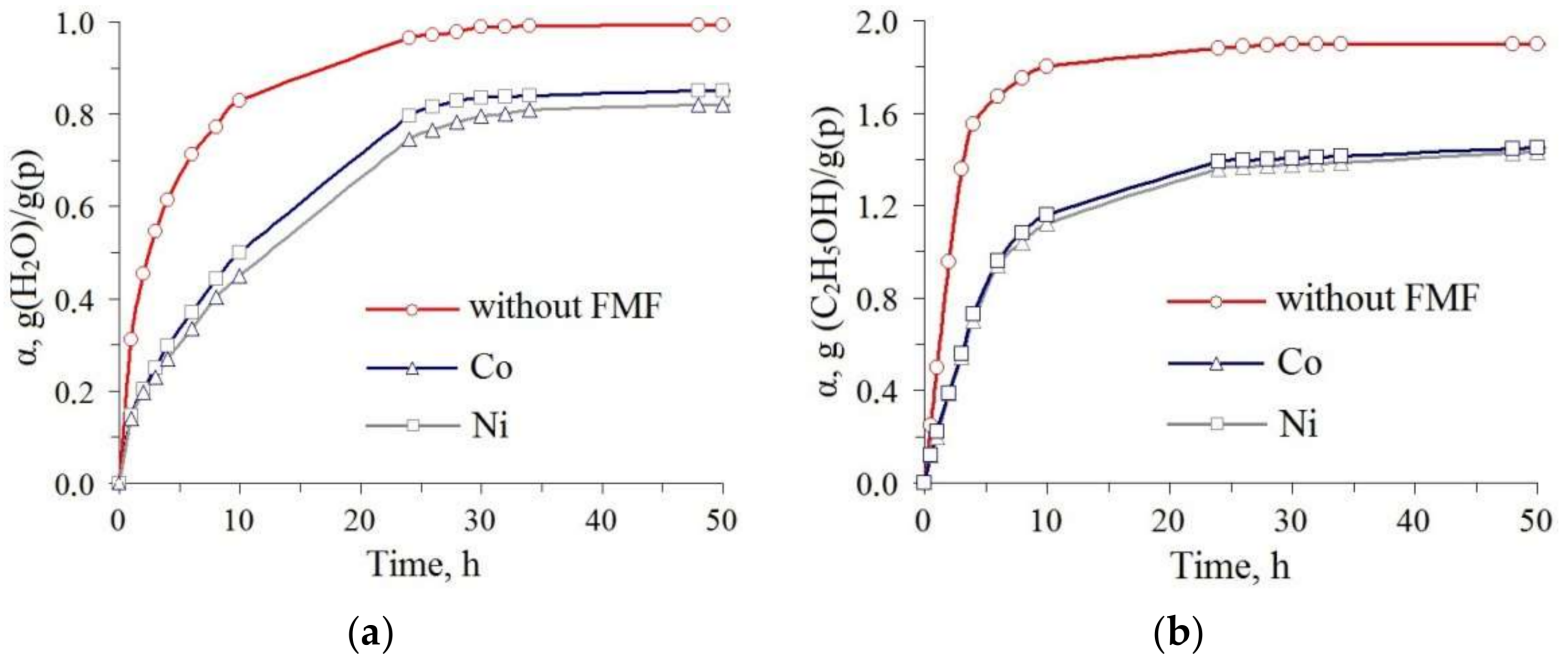
The effect of the presence of FMF in the structure of pHEMA-gr-PVP copolymers on their swelling ability (HEMA:PVP = 80:20 mass parts, [FMF] = 10 wt.%): (**a**) in water; (**b**) in ethanol.

One of the important technological characteristics of materials based on pHEMA-gr-PVP copolymers is the ability to adsorb water and other solvents, as well as to dissolve low molecular weight substances in them. The processes of interaction of polymers with low molecular weight liquids are of great importance during the synthesis of polymers, their processing and operation in various liquid media. The study of this interaction is especially important for hydrogels, being materials formed by swelling of hydrophilic copolymers during the sorption of the solvent to equilibrium. PHEMA-gr-PVP copolymers have a spatially crosslinked structure formed by blocks of HEMA grafted on PVP, and contain hydrophilic groups: hydroxyl of methacrylate and carbonyl of pyrrolidone cycle in PVP [42]. Specifically, this structure provides the sorption capacity of pHEMA-gr-PVP copolymers. Sorption capacity was characterized by swelling kinetics curves (Figure 7).

According to the research results, the introduction of FMF particles into the copolymer structure in any case led to a deterioration of the sorption capacity (Figure 7a). The shape of swelling kinetic curves is mainly affected by the structure of the polymer matrix. Since FMF particles act as additional crosslinking nodes, this led to a decrease of *M_c_* (Figure 3), i.e., an increase in the crosslinking density of the polymer network, and, accordingly, the deterioration of the swelling ability. The obtained curves for FMF/pHEMA-gr-PVP composites indicated that the equilibrium degree of swelling in distilled water was achieved for all samples over the same time. At the same time, the difference in the limit values of the degree of swelling indicated the role of the nature of the metal in the polymer matrix structure formation.

**Figure 8:**
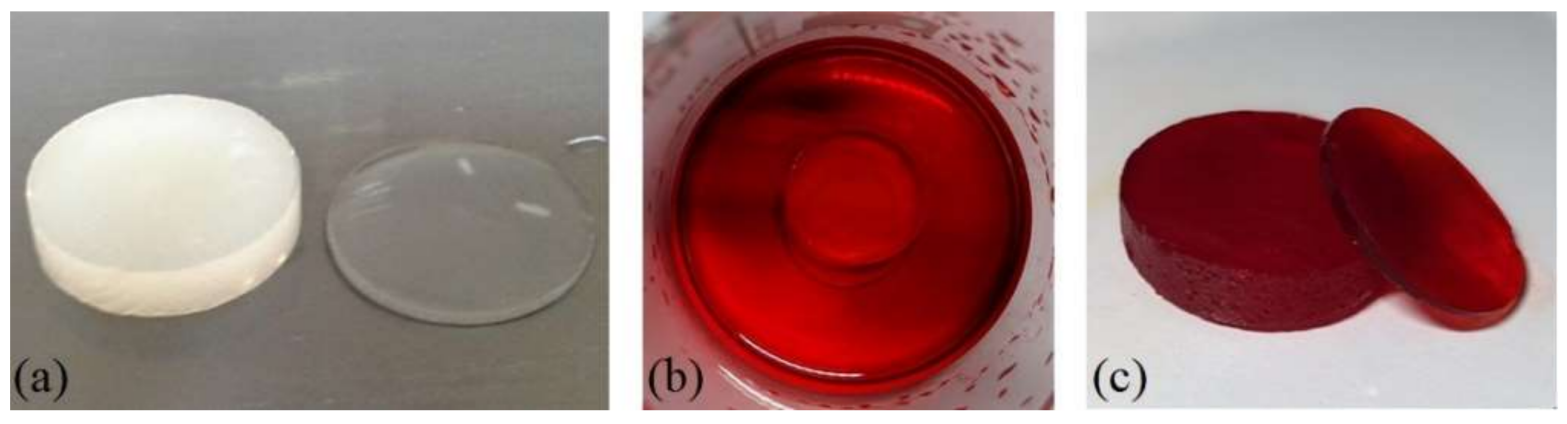
Demonstration of the possibility of sorption of organic dye by pHEMA-gr-PVP copolymer: (**a**) copolymer swollen in water; (**b**) the process of swelling of pHEMA-gr-PVP copolymer in dye solution; (**c**) pHEMA-gr-PVP copolymer with adsorbed dye.

To determine the sorption capacity of metal-filled hydrogels relative to low molecular weight substances, the behavior of the obtained materials in the medium of a less polar (relative to water) solvent, ethyl alcohol, was studied (Figure 7b). The sharp increase in the mass of samples in the ethyl alcohol medium could be explained by the intense diffusion of the latter and the higher entropy of its interaction with macro-chains. This was the result of the higher energy of the interaction of alcohol with the matrix, due to which there was a straightening of the chains of the polymer matrix with increasing distance between their individual fragments. In addition, it should be taken into account that the interaction of alcohol molecules occurs with polar groups of the copolymer such as C=O, –OH. The interaction of ethanol molecules with macromolecules of the polymer network was accompanied by blocking of polar groups in the polymer units and, consequently, distancing of network chains among themselves, which, naturally, would favor the increase of the diffusion of ethanol-dissolved low molecular weight compounds in the hydrogel volume with increasing sorption value. Together with solvents, HEMA with PVP copolymers can adsorb dissolved inorganic and organic substances in them [54,55]. In particular, the possibility of sorption of synthetic dyes of organic origin by pHEMA-gr-PVP copolymers was proved in the example of methyl red (Figure 8).

For clarity, unfilled samples of pHEMA-gr-PVP copolymers were used. Thus, the obtained FMF-filled hydrogels could be used as sorbents of inorganic and organic substances.

#### 3.2.2. Influence of Metallic Filler on Thermophysical Properties of pHEMA-gr-PVP Copolymers

**Figure 9:**
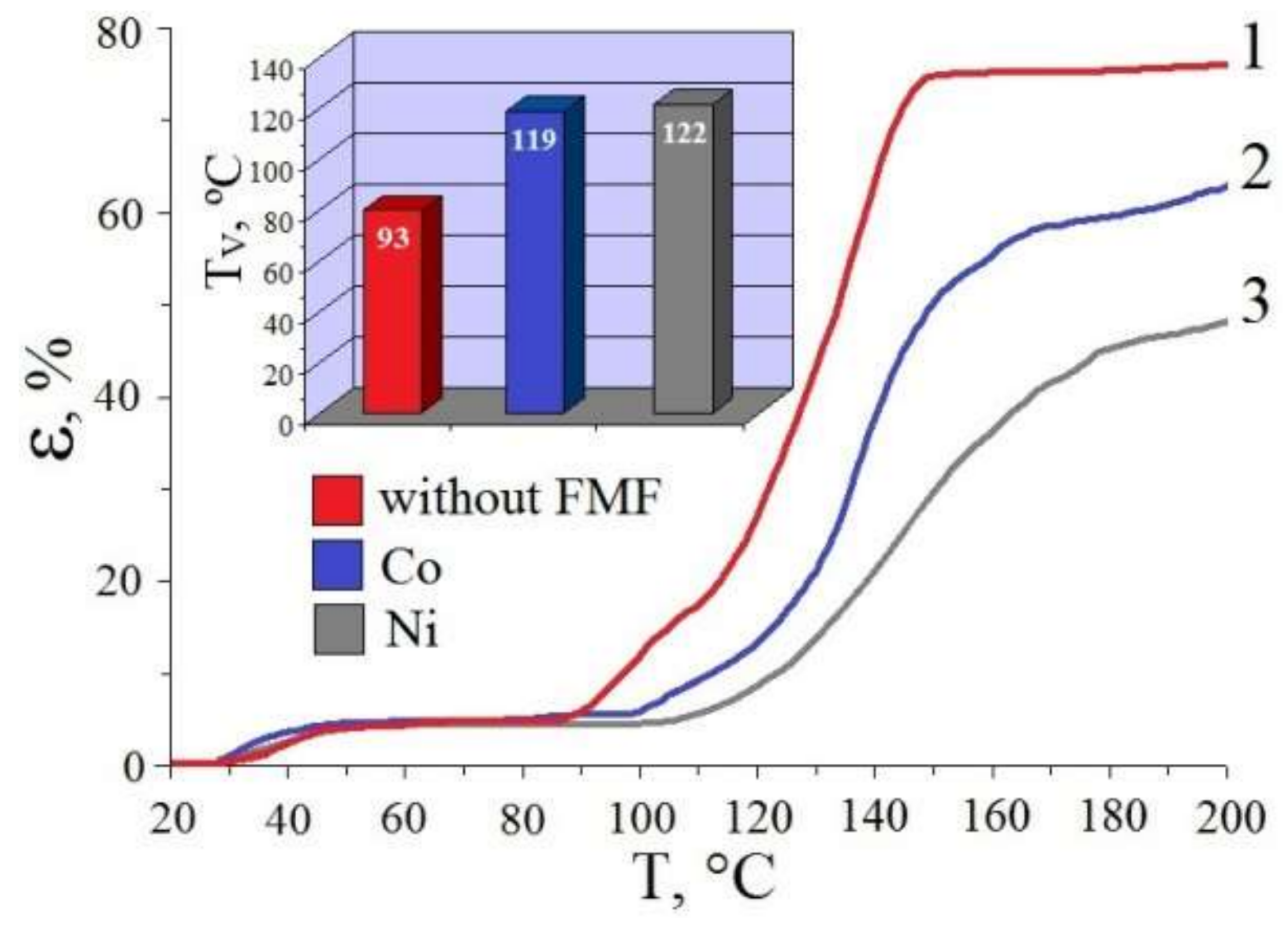
The effect of metal filler on temperature dependence of deformation (*ε*) and Vicat softening temperature (*T_V_*) of pHEMA-gr-PVP copolymers (HEMA:PVP = 80:20 mass parts, [FMF] = 10 wt.%).

Temperature intervals of phase and physical states determine the mechanical properties and, accordingly, the field of practical use of synthesized materials. Heat resistance of metal-filled composites based on pHEMA-gr-PVP copolymers was evaluated by Vicat softening temperature (*T_V_*, °C), phase transitions, using thermomechanical curves (TMCs), which represent the dependence of polymer deformation on temperature (Figure 9).

The initial section of the TMCs corresponds to the temperature region of the glassy state, where the energy of thermal motion of macromolecules is insufficient for the manifestation of deformation under load. Accordingly, for polymers only small reversible deformations at low temperatures (up to the glass transition temperature) in the glassy state are observed. The region of the curves characterized by a sharp increase in deformation in a narrow temperature range, corresponds to the structural transition of the system from a glassy state to a rubbery state. The addition of FMF to the copolymer helped to shift the glass transition temperature to the region of higher temperatures (curves 2, 3) compared to the unfilled pHEMA-gr-PVP copolymer (curve 1). During the introduction of metals into the composition, the degree of deformation of the samples decreased. Also, for each test sample there was no transition of the copolymer to a melt state, which was a confirmation of the formation of a spatially crosslinked copolymer. The addition of a metal filler to the copolymer, compared to the unfilled material, was accompanied by an increase in heat resistance (Figure 9).

#### 3.2.3. Magnetic Properties of FMF/pHEMA-gr-PVP Composites

**Figure 10:**
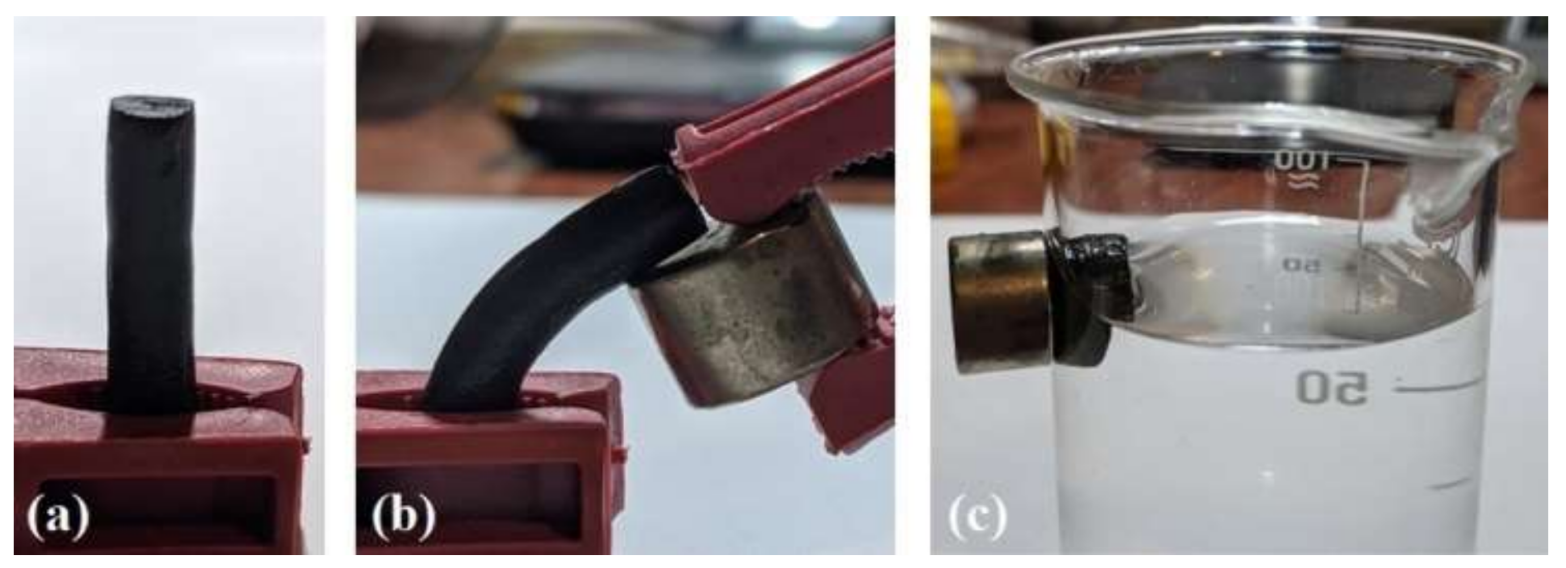
Demonstration of the hydrogel composite interaction with a constant magnet: (**a**) swollen sample based on SmCo5/HEMA-gr-PVP copolymer; (**b**) interaction of the sample with a constant magnet; (**c**) demonstration of removal of FMF-filled hydrogel from a liquid medium under the action of a constant magnet.

Sorption-capable magnetic composites and hydrogels based on them with samarium-cobalt (SmCo_5_) ferromagnet powder were obtained by polymerization with simultaneous filling of HEMA/PVP compositions (Figure 10).

**Figure 11:**
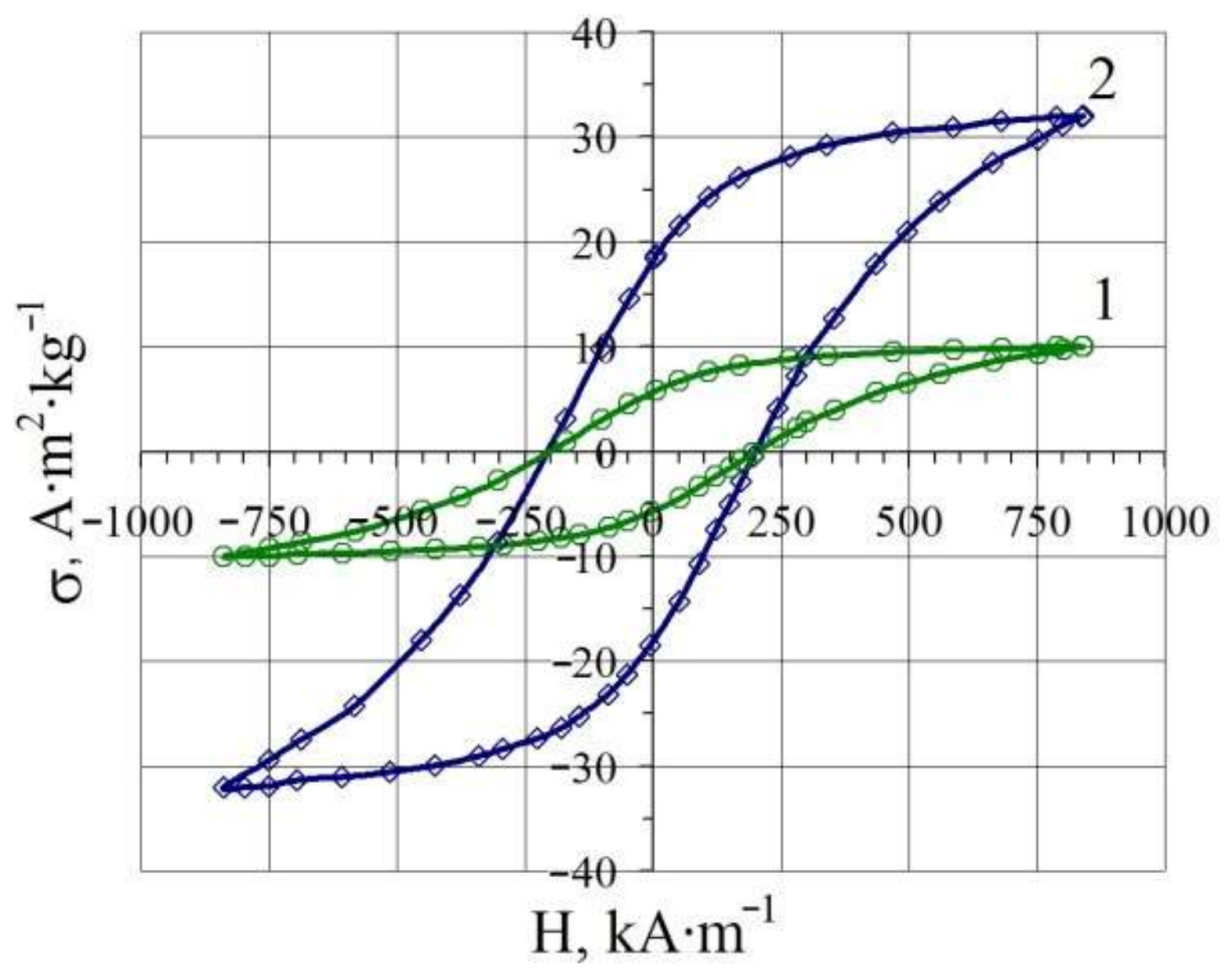
Curves of magnetization of SmCo_5_/HEMA-gr-PVP composites with different content of SmCo_5_ (Composition formulation, mass parts: HEMA:PVP = 70:30): 1—20 wt.%.; 2—80 wt.%.

SmCo_5_ was chosen as a filler due to the great attention paid to it in modern science and practice [56,57], namely due to its high coercive force, as well as resistance to corrosion and oxidation, which allows the use of composites based on it in humid and aggressive environments. It was found that SmCo_5_/HEMA-gr-PVP composites possessed magnetic properties after band magnetization using profile inductors. In order to characterize the magnetic properties of the obtained materials, their magnetization curves were studied (Figure 11), from which the value of magnetization (*σ*, A·m^2^·kg^−1^) and coercive force (*H*, kA·m^−1^) were determined.

Depending on the content of samarium-cobalt filler, hydrogel magnets were characterized by a coercive force of 200 kA × m^−1^ and induction of a magnetic field at the poles of 4–5 mT and 10–15 mT, respectively. Apparently, the particles of the ferromagnet after their introduction into the polymer matrix retained their magnetic properties. The obtained composites were characterized by the typical behavior of magnets. Filling hydrogels based on pHEMA-gr-PVP composites with magnetic fillers makes them suitable for obtaining a variety of magnetically sensitive materials with specific characteristics. For example, the combination of the magnetic properties of the filler and the sorption capacity of the hydrogel matrix is a prerequisite for the creation of currently relevant new materials, magnetic sorbents. One of the main problems associated with the usage of these kinds of adsorbents for environmental application and waste water cleaning is the removal of the adsorbents after completion of their tasks [58].

Figure 10c shows the result of the interaction of the filled hydrogel with a constant magnet. The magnetic properties of the composite ensured its unimpeded removal from the liquid medium for its regeneration and reuse.

#### 3.2.4. Electrically Conductive Properties of FMF/pHEMA-gr-PVP Composites

**Figure 12:**
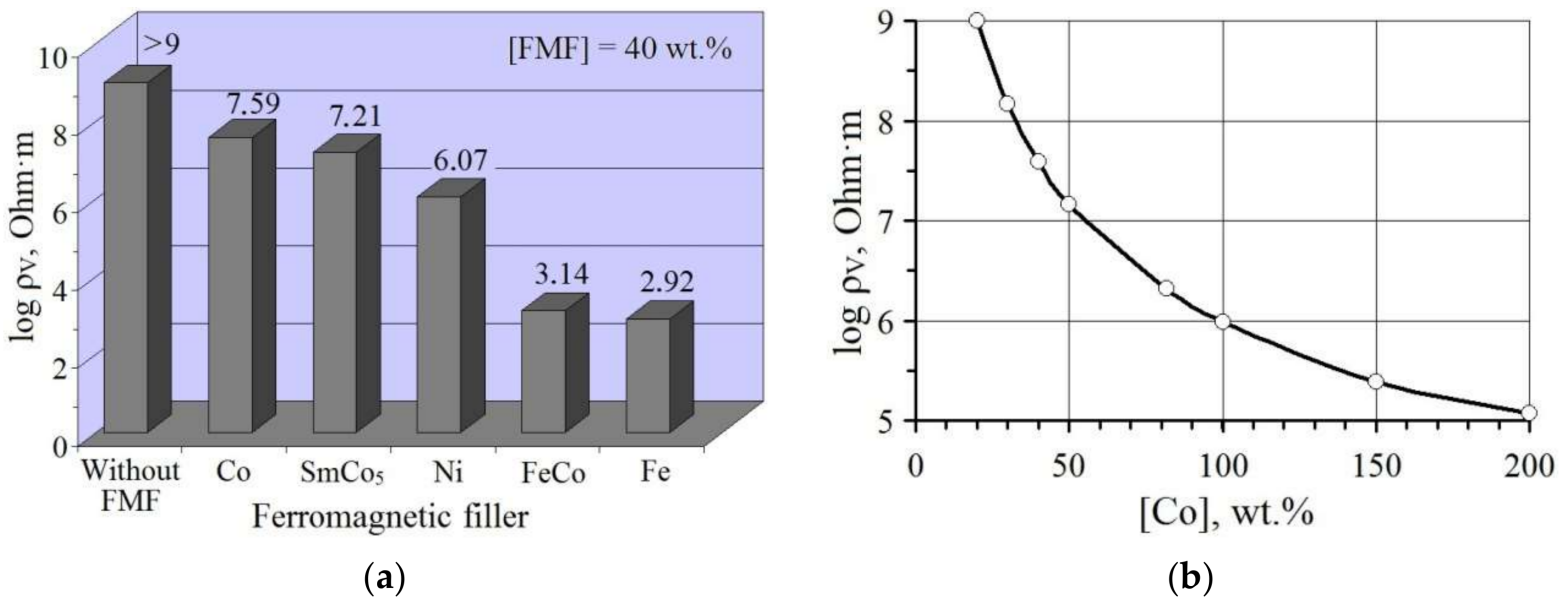
The effect of nature (**a**) and content (**b**) of FMF on electrically conductive characteristics of dry FMF/pHEMA-gr-PVP composites (HEMA:PVP = 70:30 mass parts).

The introduction of fine FMF particles in polymers, along with magnetic properties, provides them with electrical conductivity. Of particular interest to the pHEMA-gr-PVP matrix, as the object of filling, are its hydrophilicity and sorption capacity. The consequence of the synergism of the properties of the hydrophilic polymer matrix and electrically conductive filler is the ability to change the conductive characteristics of the composite depending on external factors, such as the degree of swelling, sorption of low molecular weight substances, temperature, pressure, humidity, and pH medium. The specific volumetric electrical resistance (*ρ_V_*, Ohm·m) of FMF/pHEMA-gr-PVP composites, depending on the nature of FMF particles, was investigated (Figure 12a).

**Figure 13:**
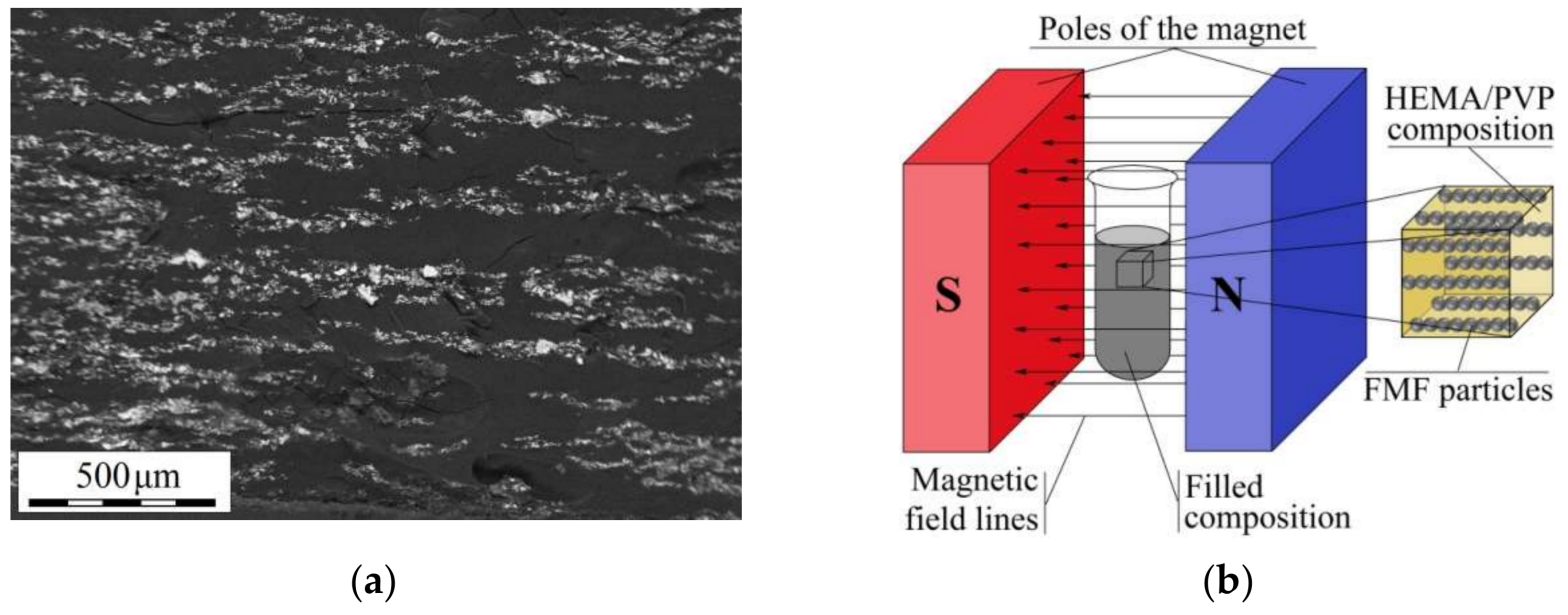
SEM micrographs of Co-filled pHEMA-gr-PVP copolymer with uniaxially ordered particles (**a**) and obtainment process scheme of a composite hydrogel in a magnetic field (**b**).

It was established that the electrical resistance of unfilled copolymers obtained by mass polymerization was characteristic for dielectrics. The appearance of electrically conductive properties in Co/pHEMA-gr-PVP composites was observed after the introduction of the filler in the amount of 20–30 wt.% into the structure of the copolymer (Figure 12b). However, as can be seen, *ρ_v_* remained quite high, even with the introduction of Co powder more than 100 wt.%. To significantly increase the electrical conductivity of polymeric FMF/pHEMA-gr-PVP composites filled with a dispersed filler, it was necessary to approach the filler particles together to direct contact or to a distance around several angstroms. In both the first and second cases, the path for the flow of electric current would be in the form of a continuous chain of contacts of sufficiently close filler particles [59]. Therefore, to increase the electrical conductivity, i.e., increase the number of contacts, it was necessary to introduce a larger amount of filler into the composition formulation. However, this method was not always technological, in that, as the weight of the sample increased, its strength characteristics deteriorated. It is also important to note that as the filler content increased, the viscosity of the original composition increased as well. For each filler there is a limit content in the composition, at which the composition still retains fluidity and, accordingly, the ability to form. With a further increase of filler content, the composition becomes non-technological. In this regard, it is of great interest to artificially obtain chain structures of electrically conductive filler in order to increase the conductive properties of polymer composites. In order to increase the electrical conductivity of FMF/pHEMA-gr-PVP composites with relatively small amounts of FMF, it was proposed to structure the filler in the composition by orienting the particles during polymerization in a constant magnetic field (Figure 13a). In a magnetic field, metal particles received an inductive magnetic moment, then, between neighboring magnetized particles, the interaction occurred and due to it they fit into continuous chains (Figure 13b).

**Figure 14:**
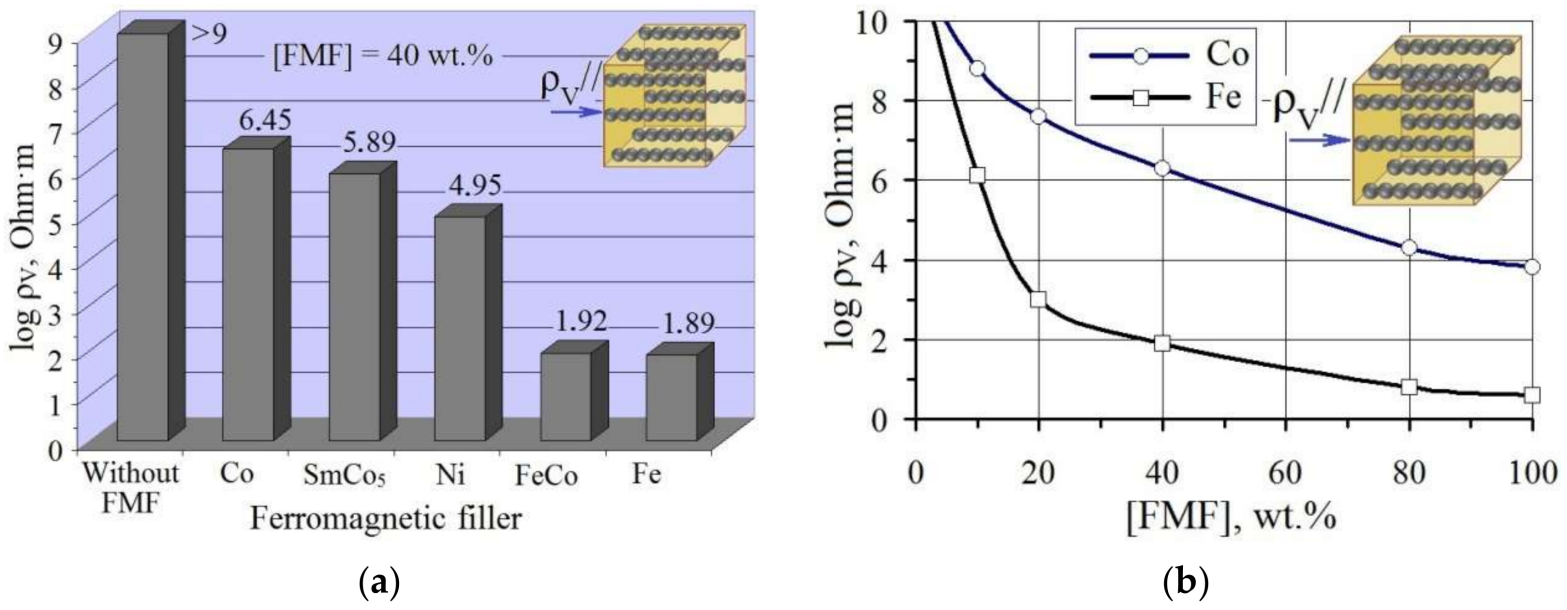
The effect of nature (**a**) and content (**b**) of FMF on volumetric electrical resistivity (*ρ_V_*) of FMF/HEMA-gr-PVP composites obtained in a magnetic field (HEMA:PVP = 70:30 mass parts, H = 23 kA/m).

In Figure 14a, the results of determining the specific resistivity *ρ_V_* for composites filled with FMF of different nature during polymerization in a magnetic field are presented. The electrical resistance of FMF/pHEMA-gr-PVP composites was significantly lower in every case, compared to unfilled pHEMA-gr-PVP copolymer, for which *ρ_V_* > 10^9^ Ohms.

At the same time, the composites obtained in this way were characterized by anisotropy of electrical conductivity, so that in the direction of the magnetic field lines, *ρ_V_//* was minimal, but in the perpendicular direction, the resistance (*ρ_V_⊥*) of composites with a filler content of 40 wt.% was greater than 10^9^ Ohms. The specific electrical resistivity of FMF/HEMA-gr-PVP composites obtained without a magnetic field (with statistical distribution of metal particles) was greater by an order (Figure 12) than *ρ_V_* materials with FMF particles oriented in a magnetic field. In Figure 14b, the results of studies characterizing the influence of FMF content on the specific resistivity value of composites, measured in the direction of chains alignment of FMF particles, are presented.

**Figure 15:**
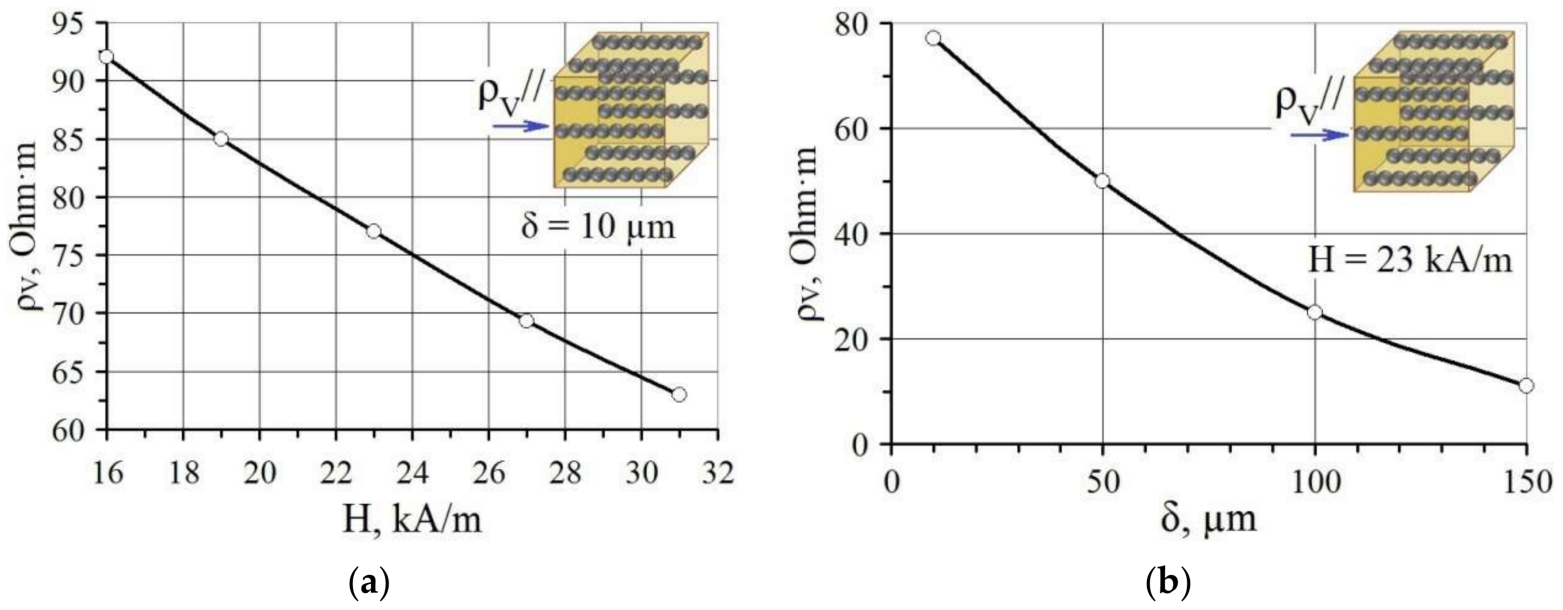
The effect of magnetic field strength (**a**) and FMF particle size (**b**) on resistivity of Fe/HEMA-gr-PVP composites (HEMA:PVP = 70:30 mass parts, [Fe] = 40 wt.%).

FMF particles under the action of a magnetic field are lined up in chains and interaction between them creates conditions for the passage of electric current. The nature of this interaction will determine the number of contacts between the filler particles, the contact strength and the thickness of the polymer film between the particles. Therefore, it could be predicted that the electrical conductivity of the composite would depend not only on the nature of the metal (magnetic and electrically conductive properties), but also on the magnetic field strength during the polymerization of the original compositions (Figure 15a).

If in the case of the distribution of FMF particles in the polymer matrix according to the statistical structure, the electrical conductivity is determined mainly by the number of particles involved in creating contacts, then in the oriented structure the main factor affecting conductivity is the contact resistance between neighboring particles and the number of contacts in a chain [60]. It is a known fact that an increase in particle size causes an increase in electrical conductivity [59]. As the particle size increased, the number of contacts decreased, and, at the same time, the effect on the conductivity of the dielectric polymer matrix decreased and the contact force between the particles increased under the action of the magnetic field, which was a prerequisite for reducing the *ρ_V_* of the composite (Figure 15b).

#### 3.2.5. Physico-Mechanical Properties

On the basis of obtained results from DMTA (Figure 5) and TMC (Figure 9) it was already possible to draw a conclusion about the significant effect of FMF on the viscoelastic and thermophysical properties of pHEMA-gr-PVP copolymers. Since the obtained materials could be operated in two physical states, glassy (hard) and rubbery (soft), so the properties were studied in both dry and hydrated samples. The influence of fine metal filler on the formation of the structure and properties of pHEMA-gr-PVP composites is determined by two factors. The first relates to the physical action on the structure of the polymer. In this regard, the role of the metal particle is, in fact, an element of hetero-phase structure. The second is the result of changes in the polymer matrix, which happen due to interaction at the interface between polymer–metal and the participation of the particle surface in the formation of chemical composition and structure of the polymer, which is manifested by the influence of filler on the grafting efficiency of PVP and *M_C_* (Figure 3 and Figure 4). The overall change in the properties of the filled system, compared with the polymer matrix, takes place due to the simultaneous action of the two factors. On the one hand, during the introduction of FMF, *M_C_* decreased (Figure 3), because the particles played the role of additional nodes of physical crosslinking. On the other hand, the presence of metals affected the efficiency of PVP grafting (Figure 3), the content of which, in the structure of the copolymer, affected its physico-mechanical and thermophysical characteristics [42].

**Figure 16:**
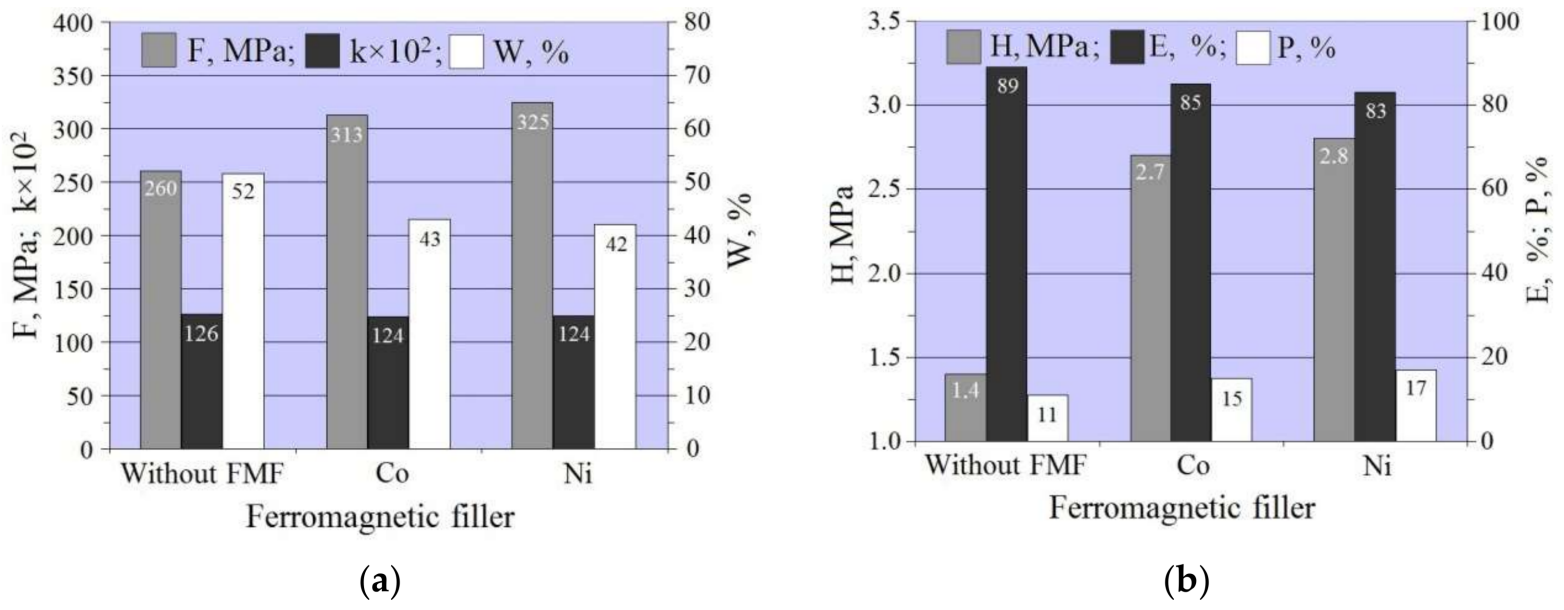
The effect of FMF presence on the physico-mechanical properties of pHEMA-gr-PVP copolymers in dry (**a**) and swollen (**b**) states (HEMA:PVP = 80:20 mass parts; [FMF] = 10 wt.%).

Metal-filled pHEMA-gr-PVP copolymers in the dry state were studied for surface hardness (*F*, MPa) and swelling ability, which was characterized by water content (*W*, %) and swelling coefficient (*k*) (Figure 16a). Copolymers, swollen in water to equilibrium, were studied as elastomers for hardness, resilience and plasticity, which were characterized, respectively, by the hardness number (*H*, MPa), the elasticity index (*E*, %) and the plasticity number (*P*, %) (Figure 16b).

**Figure 17:**
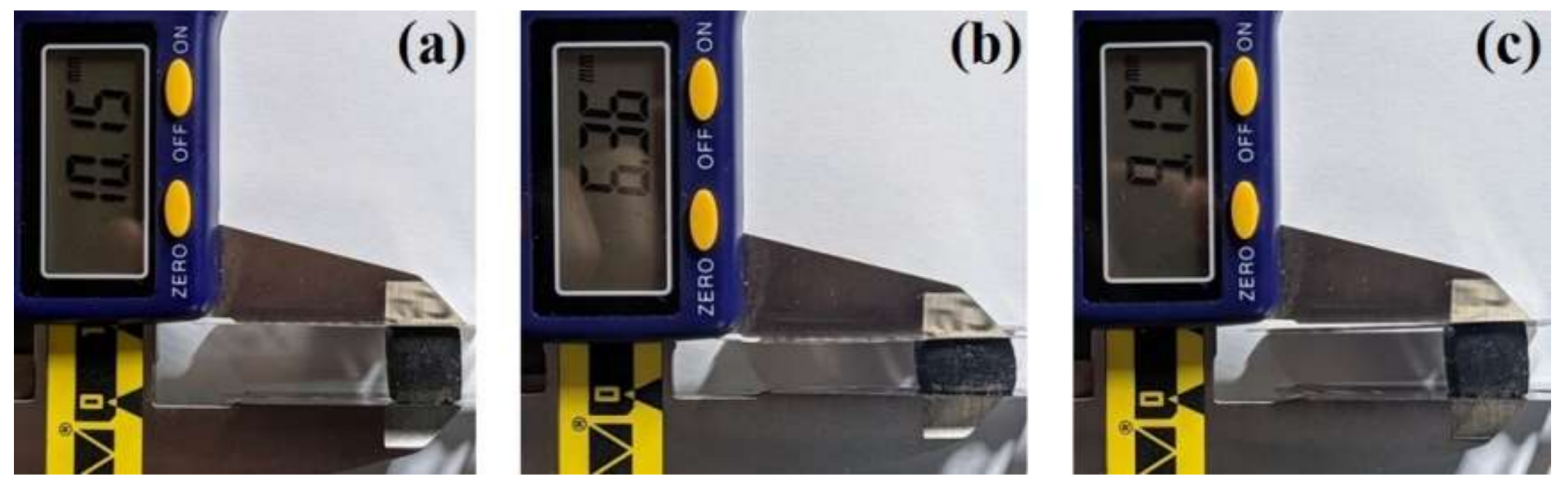
Demonstration of the resilience properties of an equilibrium swollen sample of Co-filled pHEMA-gr-PVP copolymer: (**a**) initial sample; (**b**) sample under load; (**c**) sample after unloading.

The introduction of the metal filler into the original composition formulation, in any case, compared with the unfilled material, promoted the increase of its strength characteristics. As can be seen from the presented results, the surface hardness (*F*, MPa) of dry samples with a FMF content of only 10 wt.% increased by 20–25% (Figure 16a), and the hardness number (*H*, MPa) of the samples in the swollen state doubled (Figure 16b). Obviously, the increase in the strength characteristics of composites occurred due to the increase in the degree of crosslinking and the number of physical nodes in the polymer network (Figure 3 and Figure 4b). The influence of the metal filler particles was associated with the formation of interfacial layers, the structure and properties of which differ from the characteristics of the polymer in volume (Figure 6). At the same time, individual filler particles, due to physical interaction with the components of the original polymer-monomer composition, can play the role of additional physical crosslinking nodes, compensating for the decrease in the concentration of chemical nodes. Naturally, in terms of formation of a more crosslinked polymer network, the sorption capacity of polymers decreased. The water content of composites (*W*, wt.%) decreased by 16–18% (Figure 16a). At the same time, as a result of the filling, the resilience (*E*, %) slightly decreased and the plasticity (*P*, %) increased (Figure 16b). However, the resilience properties of composites remained relatively high (Figure 17).

**Figure 18:**
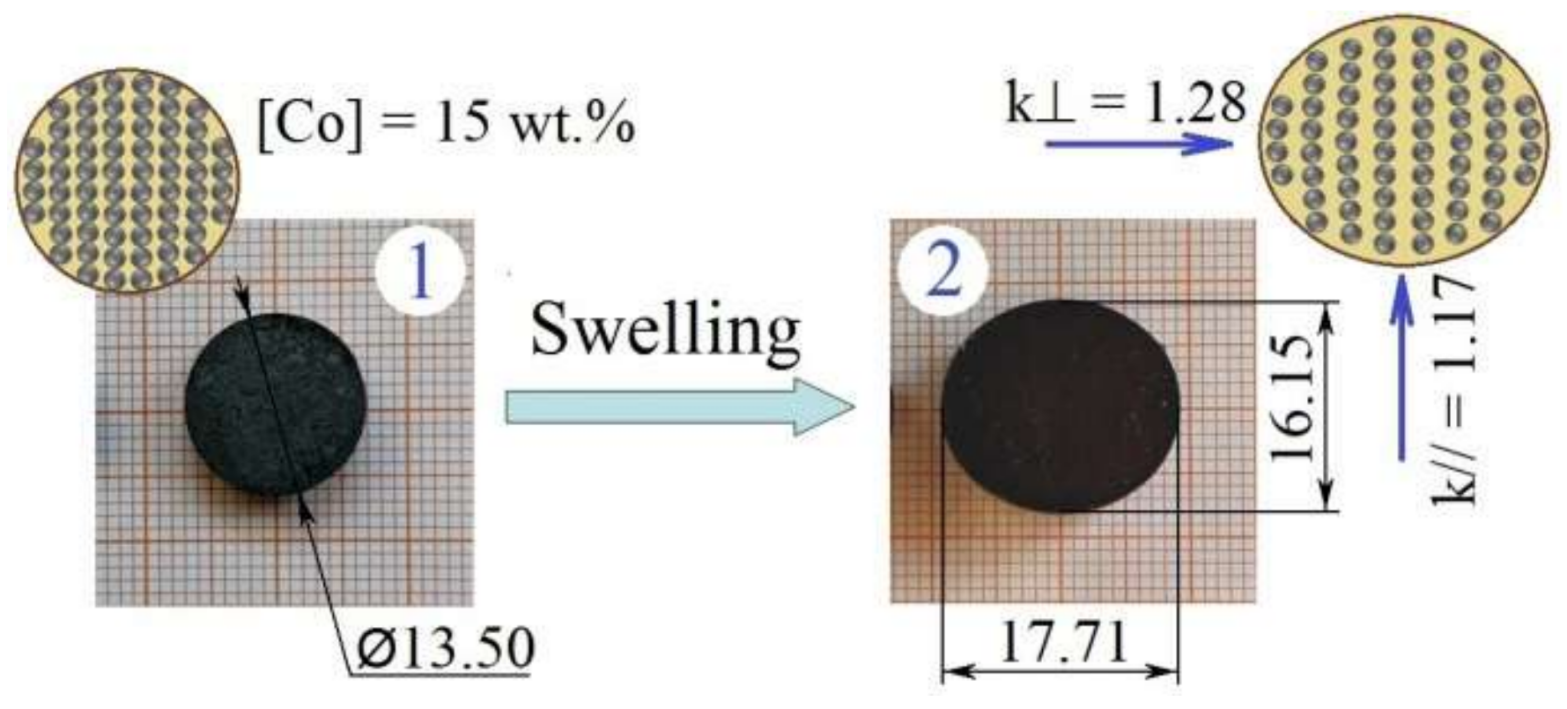
Demonstration of swelling anisotropy of a sample of Co-filled pHEMA-gr-PVP copolymer obtained by polymerization filling in a magnetic field: 1—Dry sample; 2—Equilibrium swollen sample.

During the study of the swelling process of samples of metal-filled copolymers obtained in a magnetic field (with an ordered arrangement of filler particles), the anisotropy of the swelling coefficient was detected (Figure 18).

It was established that the swelling coefficient (*k*⊥ = 1.28) in the perpendicular direction to the filler chains (in the direction of the action lines of magnetic induction forces) was greater than the swelling coefficient in the direction of aligning chains of filler particles (*k//* = 1.17).

## 4. Conclusions

Composite copolymers of HEMA with PVP, and hydrogels based on them, modified with ferromagnetic fillers (Ni, Co, Fe, FeCo, SmCo_5_), were obtained by the method of filling during polymerization.

1. It was found that the polymerization of FMF-filled compositions occurred without additional catalysts, which is the evidence of the participation of the FMF surface in initiating the polymerization process of HEMA/PVP compositions. The formation of grafted spatially crosslinked copolymer of pHEMA-gr-PVP was confirmed. The introduction of FMF particles into the original composition formulation caused the formation of copolymers with a greater degree of crosslinking, which indicates the appearance of additional spatial network due to the formation of physical nodes, the elements of which may be metal particles. It was established that in the interfacial layer, on the filler surface and in the volume, a copolymer formed with a different structure.
2. Using the example of water and ethyl alcohol, it was shown that the obtained composites are able to swell in solvents. It was established that the introduction of FMF particles into the structure of pHEMA-gr-PVP copolymers in any case led to a deterioration of the sorption capacity. At the same time, the water content decreased by 16–18% and lay within 42–43% by mass, but remained relatively high. The sorption possibility of pHEMA-gr-PVP copolymers of synthetic dyes of organic origin was shown on the example of methyl red.
3. The filling of pHEMA-gr-PVP copolymers with micro-sized FMF powders increase their heat resistance (Vicat softening temperature is 39–42 °C higher compared to the unfilled material), as well as strength characteristics (in both dry and swollen states). The surface hardness of dry samples with a FMF content of only 10 wt.% increased by 20–25%, and the hardness number of samples in a swollen state is doubled. At the same time, resilience slightly deteriorated, and so plasticity increased.
4. FMF-filled pHEMA-gr-PVP copolymers, after band magnetization with profile inductors, possess magnetic properties. It was established that the obtained composites in dry state are characterized by a coercive force of 200 kA × m^−1^ and induction of a magnetic field at the poles of 4–5 mT and 10–15 mT, respectively.
5. The introduction of fine FMF particles into pHEMA-gr-PVP copolymers (which are dielectrics in the dry state) provides them with electrical conductivity. Depending on the nature and content of FMF, the resistance of filled materials can be regulated within 10^3^–10^6^ Ohm·m. The structuring of filler particles into chains using their orientation during polymerization in a constant magnetic field provided a 10-fold increase in the electrical conductivity of composites with relatively small amounts of FMF.
6. Depending on the need and the task in hand, the research results showed, while choosing the nature of the metal-filler, it is possible to obtain hydrogel composite materials with unique predictable properties, having electrically conductive, magnetic, or sorption activities and combinations of the same. The obtained composites were not contaminated with other substances (by-products from the obtaining reaction of metal-filler), which also allowed the avoidance of an additional longtime stage of purification of the composite, washing. The high reactive capacity of the polymer-monomer composition, based on HEMA and PVP in the presence of FeSO_4_, provides the ability to obtain hydrogel materials filled with FMF at a high rate using simple technology, at room temperature, in air, without the usage of sophisticated equipment and facilities. Preliminary results indicate the prospects for the use of the obtained materials, for example, as magnetic sorbents for various applications, as well as electrically conductive materials that may respond to changes in conductivity from various factors (e.g., moisture content, differences in pressure, temperature).

## Data Availability

Not applicable.
